# RGD分子影像在肺癌的研究现状与进展

**DOI:** 10.3779/j.issn.1009-3419.2014.12.06

**Published:** 2014-12-20

**Authors:** 宁 岳, 双虎 袁, 国仁 杨

**Affiliations:** 250117 济南，山东省医学科学院，山东省肿瘤医院核医学科 Department of Nuclear Medicine, Shandong Cancer Hospital, Shandong Academy of Medical Sciences, Jinan 250117, China

**Keywords:** RGD, 整合素, 肺肿瘤, 分子影像, 血管生成, RGD, Integrin, Lung neoplasms, Molecular imaging, Angiogenesis

## Abstract

肺癌是国内外最常见、死亡率最高的恶性肿瘤之一。持续的新生血管生成是恶性肿瘤的特征，是肿瘤增殖、浸润、复发和转移的基础，也是目前肺癌生物学治疗热点之一。肿瘤血管生成过程中，整合素的作用至关重要。精氨酸-甘氨酸-天冬氨酸（Arg-Gly-Asp, RGD）肽能特异地与整合素结合，应用放射性核素标记的RGD分子探针，可使肿瘤血管显像，能反映肿瘤血管的变化。本文对近年来国内外RGD肽的肺癌显像研究进展进行综述。

## Abstract (en)

Lung cancer has been one of the most common and the highest mortality rates malignant tumors at home and abroad. Sustained angiogenesis was not only the characteristic of malignant tumors, but also the foundation of tumor proliferation, invasion, recurrence and metastasis, it was also one of the hot spots of treatments in lung cancer biology currently. Integrins played an important part in tumor angiogenesis. Arg-Gly-Asp (RGD) peptides could combine with integrins specifically, and the application of radionuclide-labeled RGD molecular probes enabled imaging of tumor blood vessels to reflect its changes. The lung cancer imaging of RGD peptides at home and abroad in recent years was reviewed in this article.

2013年中国肿瘤年报^[1]^显示，恶性肿瘤发病率第一位的是肺癌。而且肺癌的发病率和死亡率将继续快速攀升，每年死于肺癌的患者比乳腺、结肠、前列腺、胰腺癌的总和都要多。肺癌中80%是非小细胞肺癌（non-small cell lung cancer, NSCLC），术后总的5年生存率仅为20%-40%^[2]^。现阶段对于它的治疗，已经形成了一系列多学科综合治疗方案。近年发展的精氨酸-甘氨酸-天冬氨酸（Arg-Gly-Asp, RGD）示踪剂通过与肿瘤新生血管高表达的αvβ3整合素特异性结合，可以实现无创、立体、动态的血管生成显像，从而在血管生成方面显示肺癌的生物学特性。本文就RGD分子影像的肺癌研究进展进行综述。

## 1 肺癌与血管生成

血管新生是指毛细血管从已存在的血管周围生成的过程，包括细胞、可溶性因子和细胞外基质（extracellular matrix, ECM）物质间的广泛作用。1971年，Folkman提出“肿瘤的生长、侵袭和转移都依赖于肿瘤血管生成”的观点。不断扩张的新生血管网为肿瘤细胞提供生存所必需的营养并清除其代谢产物，如果血管生成受阻，没有足够血供，肿瘤的生长将不会超过3 mm。因此，肿瘤被认为是一种血管生成依赖性疾病。在肿瘤的发生发展过程中，持续无规则的新血管生成加速了疾病发展。

肿瘤区血管密度可达正常组织血管密度的50倍-200倍，且在结构、功能以及分子水平上与正常血管存在明显差异^[3]^，临床上高密度血管生成的肿瘤患者预后一般较差。肺癌是典型的血管依赖性病变，与其他实体肿瘤一样，血管的生成与肺癌的发生、生长及转移密切相关。目前已分离和纯化了20多种血管生成因子，其中血管内皮生长因子/受体（vascular endothelial growth factor/VEGF receptors, VEGF/VEGFRs）及其他相关的生物信号分子、蛋白质（如αvβ3、Endoglin-CD105等）在肺癌血管生成中具有重要的、乃至中枢核心的调节作用，已经成为肺癌诊断与治疗的重要靶点^[4]^。

## 2 整合素αvβ3与RGD肽

整合素为细胞黏附分子家族的重要成员之一，是一组广泛存在的跨膜糖蛋白，此异二聚体由一个α链和一个β链以非共价键结合而成。迄今为止已发现20种不同的α链（120 kDa-185 kDa）和11种不同的β链（90 kDa-110 kDa），至少组成20余种不同的整合素亚型。其中，αvβ3、αvβ5、αvβ1与RGD序列的关系最密切。

20世纪80年代初期，人们从ECM中分离出大量的糖蛋白。大多数ECM糖蛋白在细胞粘附中起着重要作用，因此，称为粘附蛋白。不少粘附蛋白分子中含有RGD三肽序列。自从1984年Pierschbacher和Ruosluherci首次报道纤维蛋白原中所含的RGD三肽序列为细胞识别位点以来，RGD肽及其衍生物就成为各国学者关注与研究的热点之一。RGD短肽广泛存在于生物体内，具有分子量小、受分散有关的剪切力和有机溶剂影响较小的优势，是整合素与其配体蛋白相互作用的识别位点，介导细胞与胞外基质及细胞间的粘附作用，同时具有信号传导功能，从而介导许多重要的生命活动。

整合素在正常血管内皮和上皮细胞很少表达，但在肺癌、骨肉瘤、成神经细胞瘤、乳腺癌、前列腺癌、膀胱癌、胶质母细胞瘤及浸润性黑色素瘤等多种实体肿瘤细胞表面有高水平的表达，而且在所有肿瘤组织新生血管内皮细胞膜有高表达^[5]^，提示整合素αvβ3在肿瘤生长、侵袭和转移过程中起着关键作用。与正常组织相比，肿瘤细胞或肿瘤微环境中高表达的受体，为放射性标记的受体特异性肽非侵入性地发现肿瘤提供了分子基础。因此，放射性标记的整合素αvβ3特异性靶向RGD肽，对肺癌进行核医学显像，对其早期发现和非侵入性地监测肺癌血管生成的状态有很大的潜力，能够达到早期诊断和治疗目的。

在多种针对血管为靶点的肿瘤治疗中，整合素αvβ3受到广泛的关注。测定肿瘤组织中整合素αvβ3受体的表达水平，有助于评价肿瘤的生长状况和侵袭性。研究表明，肿瘤细胞αvβ3的表达与恶性肿瘤的浸润转移能力等恶性表型有关^[6]^，整合素αvβ3的表达水平可以作为判断一些恶性肿瘤的预后指标^[7]^。

## 3 RGD血管生成分子影像

对肿瘤血管生成状态的有效评估能够反映组织微环境状况及生物学形态，评价肿瘤组织对治疗的反应。以往认为微血管密度（microvessel density, MVD）计数是评价肿瘤内血管生成情况的“金标准”，它是肿瘤标本免疫组化染色后测定的新生血管内皮细胞数目。但其属于创伤性检查，并且受到取材部位的影响，不能反映活体血管情况及抑制肿瘤血管生成的疗效^[8]^，加上其测定方法复杂，使得MVD计数在临床应用中有较大局限性。

近年来快速发展的分子影像学成像方式在很大程度上克服了这些缺点，它将传统显像技术和新的分子显像探针结合，可监测不同阶段肿瘤发展，是一种能在活体快速、实时、无创、准确反映肿瘤全貌的影像学方法。肿瘤血管生成的显像以及抗肿瘤血管生成治疗效果的评价为其研究的重点，同时，既往的相关研究^[9]^也从一定程度上证实了肺癌的RGD血管生成显像与免疫组化染色及放射自显影有着良好的相关性。

利用放射性核素标记的RGD多肽作为整合素αvβ3的分子探针，是近些年来核医学研究的热点，已应用于肿瘤显像与治疗研究。放射性核素标记的RGD示踪剂与肿瘤新生血管高表达的αvβ3整合素的靶向显像，是目前应用前景最好的肿瘤血管生成分子影像学方法。

## 4 RGD分子影像在肺癌中的应用

目前为止，用不同的放射性核素对RGD肽进行标记的方法已经基本成熟，在正常动物体内验证了其良好的生物学分布和肿瘤靶向特性^[10, 11]^。此外，多种放射性核素标记的RGD肽已被证实为应用前景良好的αvβ3受体显像剂，如^64^Cu、^18^F、^99m^Tc、^125^I、^188^Re、^111^In和^90^Y等，已经在多种荷瘤动物模型上获得成功^[12-15]^。在临床方面，^18^F-Galacto-RGD已成为第一个进入临床试验的非侵入的整合素αvβ3靶向肿瘤显像剂，成功地应用于肿瘤患者的PET诊断，在胶质母细胞瘤的临床试验^[16]^中表现出好的生物学分布及特异性靶点识别。

### 4.1 RGD分子影像在肺癌的实验研究

在过去的几十年中，一系列基于肽的成像剂，已经被开发和研究关于各种肿瘤的靶向受体成像，包括肺癌^[17, 18]^。随着分子影像学技术的发展，^18^F-脱氧葡萄糖（fluorodeoxyglucose, FDG）PET/CT已经证明是NSCLC诊断、分期和指导治疗的准确和必要的方法^[19, 20]^。其能准确反映体内器官和组织对葡萄糖的代谢水平，应用较为广泛。但一些局部或全身感染性病灶、非特异性炎性组织以及一些良性肿瘤可以不同程度的摄取^18^F-FDG，易出现假阳性结果，对肿瘤诊断缺乏明显的特异性和高选择性^[21]^。而RGD整合素受体显像具有组织特异性好、亲和力和选择性高、血液清除快、T/NT值高、图像质量好等优点，并且能准确、动态、实时地反映肿瘤血管生成的变化，是具有高灵敏性与高特异性的肿瘤显像剂。

有文献^[22, 23]^表明，通过^99m^Tc-RGD-4CK在健康动物体内的药代动力学、生物分布特点及显像表现，证明其体内稳定性高，具有良好的放射化学性质及比较理想的体内动力学。且其非靶组织本底低，图像质量好，是非常有前景的肺部肿瘤阳性显像剂。^99m^Tc-RGD-4CK组小鼠的显像和生物分布T/NT值明显高于^18^F-FDG组，表明^99m^Tc-RGD-4CK对NCI-H358人NSCLC的显像敏感性高于^18^F-FDG，在诊断低代谢的NSCLC时，^99m^Tc-RGD-4CK可能比^18^F-FDG更具优势。并且在NCI-H358异种移植裸鼠提供的证据^[24]^表明，^99m^Tc-RGD-4CK主要经肾脏快速排泄，是一种很有前途的显像剂，可以无创测定细支气管肺泡癌中αvβ3整合状态和疗效监测。

检测癌症的双受体靶向分子显像剂的开发和应用一直在得到越来越多的关注^[25]^。其中，^99m^Tc-HYNIC（tricine）（TPPTS）-RGD-BBN（^99m^Tc-RGD-BBN）的生物分布、平面γ成像和小动物SPECT/CT研究被用于Lewis肺癌（LLC）C57/BL6小鼠。研究结果^[26]^表明，^99m^Tc-RGD-BBN在区分肺癌和炎症方面优于^18^F-FDG。^99m^Tc-RGD-BBN的小动物SPECT/CT可明确检测肺转移，^99m^Tc-RGD-BBN的SPECT/CT将为无创发现肺癌提供一个有效的方法。

研究^[27]^对^18^F-FB-RGD、^18^F-FDG在Lewis肺癌小鼠模型进行对比实验研究，在^18^F-FB-RGD的生物分布研究中，^18^F-FB-RGD在肿瘤部位相当高的放射性摄取，血液清除速率很快，肿瘤对血、肌肉及肺的T/NT比值均大于2.0，对原发肿瘤和对侧肺转移瘤都有清晰显像；而^18^F-FDG则由于心脏高摄取及肺部的高本底而无法区分肿瘤与纵隔，特异性不强，而且未发现转移瘤，灵敏度较^18^F-FB-RGD差。结果表明，^18^F-FB-RGD是一种对肺癌具有良好特异性及敏感性的肿瘤受体显影示踪剂，有望在肺癌诊断、分期、疗效监测方面发挥作用。

研究^[28]^表明，通过应用^64^Cu-DOTA-PEG-E[C（RGDyK）]2在肺癌小鼠模型成像，证明^64^Cu-DOTA-PEGE[C（RGDyK）]2是一个对整合素αvβ3阳性肿瘤显像很好的正电子发射断层扫描（positron emission tomography, PET）示踪剂。其主要经过肾脏从血液中快速清除，在正常肺组织和心脏最小程度的非特异性活性的积累，呈现高品质的原位肺癌图像。RGD PET显像肺癌原发灶边界的效果与FDG PET类似，显像纵隔淋巴结转移和对侧肺转移灶的效果更好。

Decristoforo等^[29]^报道用^99m^Tc标记HYNIC与c（RGDyK）组成的共轭物^99m^Tc-EDDA/HYNIC-RGD，正常小鼠和荷非小细胞肺肿瘤、荷人黑色素瘤裸鼠对其生物分布进行评价。实验证明其在体内外均有较好的水溶性、稳定性、较为理想的药代动力学及特异性蛋白结合特性，是一个很有发展前景的αvβ3受体显像剂。肿瘤摄取的研究表明，与[^18^F]Galacto-RGD相比，对αvβ3受体阳性的肿瘤其具有特异靶向性及较好的肿瘤-器官比率。

胡四龙等^[30]^探讨^99m^Tc标记RGD小分子多肽GY11作为肿瘤显像剂的可能性，并建立肺癌H460、荷人黑色素瘤A375和宫颈癌Hela BALB/a裸鼠肿瘤模型，分别进行体内分布和肿瘤显像研究。结果表明^99m^Tc-GY11主要经过肾脏从血液清除，除宫颈癌Hela肿瘤显像不明显外，给药2 h后肺癌细胞H460和黑色素瘤细胞A375肿瘤均能清除显像，24 h后显像更清晰。实验表明其有望成为肿瘤αvβ3受体显像剂。

Jung等^[31]^用^99m^Tc标记糖化的RGD肽[^99m^Tc-D-c（RGDfK）]，在Lewis肺癌、荷纤维肉瘤小鼠进行体内分布和显像研究，并在个别Lewis肺癌小鼠进行紫杉醇抗血管生成治疗。^99m^Tc-D-c（RGDfK）在血液中迅速清除，肿瘤呈高度摄取，显像过程中肿瘤清晰可见。经紫杉醇治疗的小鼠表现出对肿瘤生长速度减慢的剂量依赖性，这一现象可以用与整合素表达水平明显相关的肿瘤部位对^99m^Tc-D-c（RGDfK）的摄取降低来解释。实验结果表明，^99m^Tc-D-c（RGDfK）在体内具有良好的生物动力学及肿瘤靶向特性，并且可能在肿瘤整合素表达的非侵入评价及抗血管生成治疗的反应上起到一定作用。

### 4.2 RGD分子影像在肺癌的临床研究

Chin等^[10]^在健康人的实验报告，[^18^F]FPP（RGD）2主要通过肾脏和膀胱排出体外，在肠、甲状腺和脑室中可见少量放射性摄取，而在头、颈、胸部和四肢未见明显的放射性摄取，且在肺脏的本底非常低，因此，有望成功检测肺部肿瘤生长和转移变化。临床试验表明，RGD PET安全耐受，且无毒副作用发生。

为了进一步评价^18^F-FPPRGD2示踪剂的安全性，生物分布和剂量学特性，Mittra等^[32]^在5位健康志愿者进行PET显像，在研究过程中无不良事件发生，其有理想的药代动力学和生物分布属性，平均有效剂量为（0.146, 2±0.066, 9）rem/mCi。结论表明，^18^F-FPPRGD2示踪剂有多种应用潜力，主要作用是有望对肿瘤进行PET显像评估，由于其生物分布在头、颈和胸部的低背景摄取，对这些部位的恶性肿瘤（如脑癌、乳腺癌或肺癌等）评估是最优化的。^18^F-FPPRGD2扫描还可用于识别抗血管生成药物治疗的恶性肿瘤，有望更精确地在早期评估治疗反应。最终，涉及癌症患者的进一步的临床研究仍是必需的，以确定其特异性及可达到的肿瘤-背景比。作者下一步的计划是监测该示踪剂在脑癌、肺癌、乳腺癌患者肿瘤分期的有效性及抗血管生成治疗的反应。最近被批准用于临床研究，然而，费时多步合成限制了其临床广泛应用。

在另一项研究^[33]^中，开发了一种简单的冻干试剂盒用于标记PRGD2肽（^18^F-ALF-NOTA-PRGD2，记为^18^F-alfatide），并对9例肺癌患者进行^18^F-alfatide静态和动态PET显像。结论表明，具有良好的放射化学产率和纯度的^18^F-alfatide可以通过一个简单的冻干试剂盒一步生产，^18^F-alfatide PET允许肺癌的具有良好对比度的αvβ3成像。但是仍需要更多^18^F-alfatide的临床试验研究，来进一步证明未来结合化疗或放疗的抗血管生成疗法的可行性。

NC100692是包含单体RGD三肽序列的环状肽，Axelsson等^[34]^为了验证其^99m^Tc标记物^99m^Tc-NC100692检测肺癌转移灶的可能性，并评估其安全性，在15例肺癌患者进行全身骨显像及SPECT/CT显像。结果表明，^99m^Tc-NC100692在检测肺癌患者的肺、脑等软组织转移灶是可行的，而肝和骨病灶的检出率较差。同时证明使用^99m^Tc-NC100692是安全的，其耐受性良好。

一项旨在探讨^99m^Tc-3PRGD2对肺癌患者诊断、评估效能的多中心研究^[35]^表明，在70例肺部良恶性病变的患者进行全身平面扫描和胸部SPECT/CT，^99m^Tc-3PRGD2的成像在1 h对肺癌检测敏感，大多数恶性肿瘤表现突出的T/B比率，半定量分析的敏感性为88%，然而特异性只有58%-67%。可视化分析可辅助半定量评估，全身平面扫描和胸部SPECT/CT对原发肿瘤和转移灶的评估是互补的。这项研究的初步结果表明，^99m^Tc-3PRGD2作为整合αvβ3受体显像的一种新型示踪剂，值得进一步临床研究。

总之，RGD血管生成显像是目前较为理想的血管成像个体化评价方法，融合2种或2种以上成像方式，寻求多模式、多技术的成像方式，从结构、功能和分子水平来更好的评价治疗前后肿瘤血管的状况，将是肿瘤血管生成成像发展的重要趋势^[36]^。

## 5 小结

放射性核素标记的RGD分子探针，作为特异的靶向受体显像剂，可实现无创、立体、动态的血管生成实时显像，已用于多种肿瘤的研究。动物试验研究和临床研究初步证明其显示αvβ3受体阳性肿瘤的可能性，但是尚需更为深入的临床前与临床研究。理论上，RGD分子影像可在肺癌诊断、靶向治疗及放射治疗中发挥作用，可用于即时或超早期评估疗效、预测预后，特别是在肺癌个体化治疗方面具有广阔的临床应用前景。
